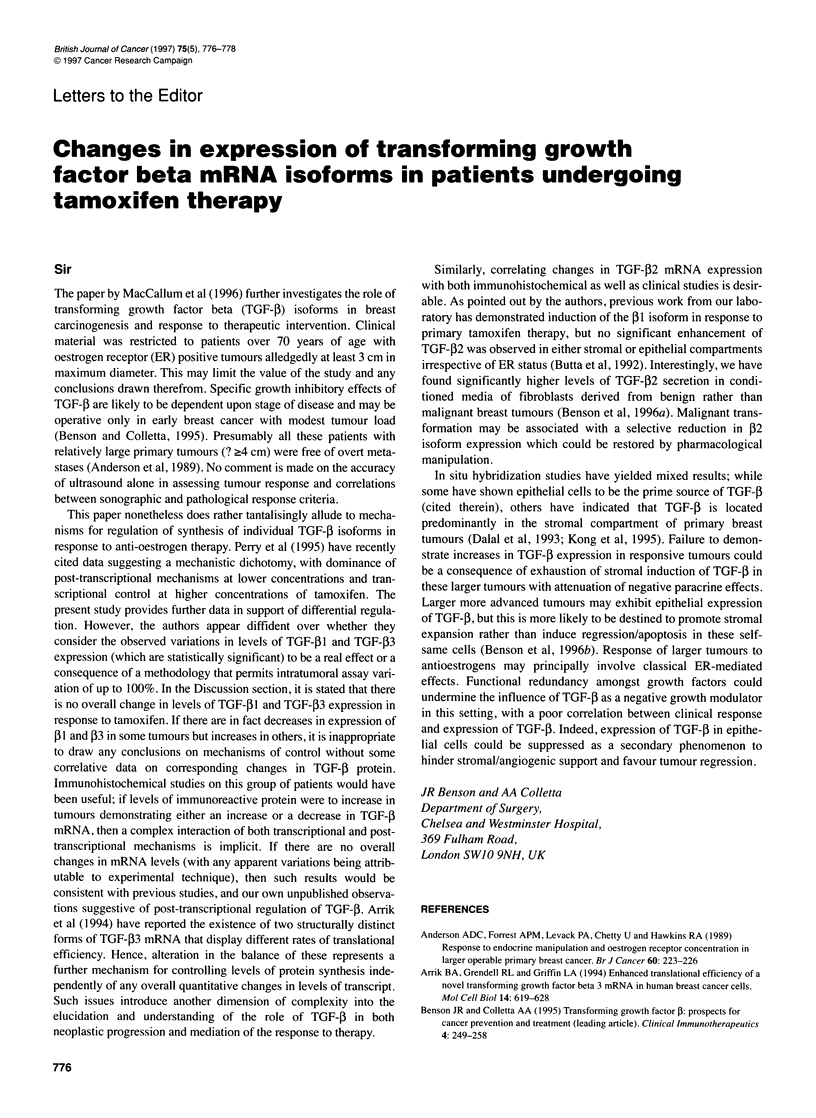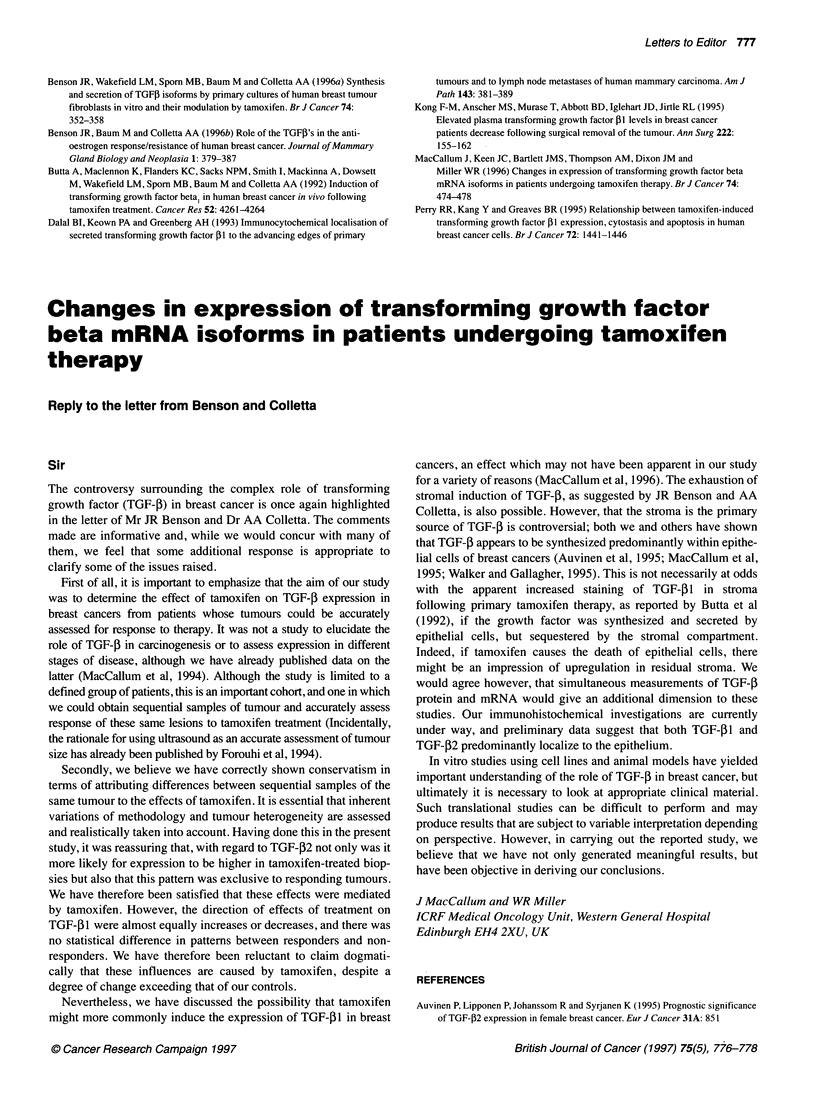# Changes in expression of transforming growth factor beta mRNA isoforms in patients undergoing tamoxifen therapy.

**DOI:** 10.1038/bjc.1997.138

**Published:** 1997

**Authors:** J. R. Benson, A. A. Colletta


British Journal of Cancer (1997) 75(5), 776–778
© 1997 Cancer Research Campaign

### Letters to the Editor

### Changes in expression of transforming growth factor beta mRNA isoforms in patients undergoing tamoxifen therapy

### Sir

The paper by MacCallum et al (1996) further investigates the role of transforming growth factor beta (TGF-β) isoforms in breast carcinogenesis and response to therapeutic intervention. Clinical material was restricted to patients over 70 years of age with oestrogen receptor (ER) positive tumours alledgedly at least 3 cm in maximum diameter. This may limit the value of the study and any conclusions drawn therefrom. Specific growth inhibitory effects of TGF-β are likely to be dependent upon stage of disease and may be operative only in early breast cancer with modest tumour load (Benson and Colletta, 1995). Presumably all these patients with relatively large primary tumours (? ≥4 cm) were free of overt metastases (Anderson et al, 1989). No comment is made on the accuracy of ultrasound alone in assessing tumour response and correlations between sonographic and pathological response criteria.

This paper nonetheless does rather tantalisingly allude to mechanisms for regulation of synthesis of individual TGF-β isoforms in response to anti-oestrogen therapy. Perry et al (1995) have recently cited data suggesting a mechanistic dichotomy, with dominance of post-transcriptional mechanisms at lower concentrations and transcriptional control at higher concentrations of tamoxifen. The present study provides further data in support of differential regulation. However, the authors appear diffident over whether they consider the observed variations in levels of TGF-β1 and TGF-β3 expression (which are statistically significant) to be a real effect or a consequence of a methodology that permits intratumoral assay variation of up to 100%. In the Discussion section, it is stated that there is no overall change in levels of TGF-β1 and TGF-β3 expression in response to tamoxifen. If there are in fact decreases in expression of β1 and β3 in some tumours but increases in others, it is inappropriate to draw any conclusions on mechanisms of control without some correlative data on corresponding changes in TGF-β protein. Immunohistochemical studies on this group of patients would have been useful; if levels of immunoreactive protein were to increase in tumours demonstrating either an increase or a decrease in TGF-β mRNA, then a complex interaction of both transcriptional and post-transcriptional mechanisms is implicit. If there are no overall changes in mRNA levels (with any apparent variations being attributable to experimental technique), then such results would be consistent with previous studies, and our own unpublished observations suggestive of post-transcriptional regulation of TGF-β. Arrik et al (1994) have reported the existence of two structurally distinct forms of TGF-β3 mRNA that display different rates of translational efficiency. Hence, alteration in the balance of these represents a further mechanism for controlling levels of protein synthesis independently of any overall quantitative changes in levels of transcript. Such issues introduce another dimension of complexity into the elucidation and understanding of the role of TGF-β in both neoplastic progression and mediation of the response to therapy.

Similarly, correlating changes in TGF-β2 mRNA expression with both immunohistochemical as well as clinical studies is desirable. As pointed out by the authors, previous work from our laboratory has demonstrated induction of the β1 isoform in response to primary tamoxifen therapy, but no significant enhancement of TGF-β2 was observed in either stromal or epithelial compartments irrespective of ER status (Butta et al, 1992). Interestingly, we have found significantly higher levels of TGF-β2 secretion in conditioned media of fibroblasts derived from benign rather than malignant breast tumours (Benson et al, 1996*a*). Malignant transformation may be associated with a selective reduction in β2 isoform expression which could be restored by pharmacological manipulation.

In situ hybridization studies have yielded mixed results; while some have shown epithelial cells to be the prime source of TGF-β (cited therein), others have indicated that TGF-β is located predominantly in the stromal compartment of primary breast tumours (Dalal et al, 1993; Kong et al, 1995). Failure to demonstrate increases in TGF-β expression in responsive tumours could be a consequence of exhaustion of stromal induction of TGF-β in these larger tumours with attenuation of negative paracrine effects. Larger more advanced tumours may exhibit epithelial expression of TGF-β, but this is more likely to be destined to promote stromal expansion rather than induce regression/apoptosis in these self-same cells (Benson et al, 1996*b*). Response of larger tumours to antioestrogens may principally involve classical ER-mediated effects. Functional redundancy amongst growth factors could undermine the influence of TGF-β as a negative growth modulator in this setting, with a poor correlation between clinical response and expression of TGF-β. Indeed, expression of TGF-β in epithelial cells could be suppressed as a secondary phenomenon to hinder stromal/angiogenic support and favour tumour regression.

*JR Benson and AA Colletta*
*Department of Surgery,*
*Chelsea and Westminster Hospital,*
*369 Fulham Road,*
*London SW10 9NH, UK*

### REFERENCES

Anderson ADC, Forrest APM, Levack PA, Chetty U and Hawkins RA (1989) Response to endocrine manipulation and oestrogen receptor concentration in larger operable primary breast cancer. *Br J Cancer* **60**: 223–226

Arrik BA, Grendell RL and Griffin LA (1994) Enhanced translational efficiency of a novel transforming growth factor beta 3 mRNA in human breast cancer cells. *Mol Cell Biol* **14**: 619–628

Benson JR and Colletta AA (1995) Transforming growth factor β: prospects for cancer prevention and treatment (leading article). *Clinical Immunotherapeutics* **4**: 249–258

Benson JR, Wakefield LM, Sporn MB, Baum M and Colletta AA (1996*a*) Synthesis and secretion of TGFβ isoforms by primary cultures of human breast tumour fibroblasts in vitro and their modulation by tamoxifen. *Br J Cancer* **74**: 352–358

Benson JR, Baum M and Colletta AA (1996*b*) Role of the TGFβ's in the anti-oestrogen response/resistance of human breast cancer. *Journal of Mammary Gland Biology and Neoplasia* **1**: 379–387

Butta A, Maclennon K, Flanders KC, Sacks NPM, Smith I, Mackinna A, Dowsett M, Wakefield LM, Sporn MB, Baum M and Colletta AA (1992) Induction of transforming growth factor $beta_1$ in human breast cancer *in vivo* following tamoxifen treatment. *Cancer Res* **52**: 4261–4264

Dalal BI, Keown PA and Greenberg AH (1993) Immunocytochemical localisation of secreted transforming growth factor β1 to the advancing edges of primary tumours and to lymph node metastases of human mammary carcinoma. *Am J Path* **143**: 381–389

Kong F-M, Anscher MS, Murase T, Abbott BD, Iglehart JD, Jirtle RL (1995) Elevated plasma transforming growth factor β1 levels in breast cancer patients decrease following surgical removal of the tumour. *Ann Surg* **222**: 155–162

MacCallum J, Keen JC, Bartlett JMS, Thompson AM, Dixon JM and Miller WR (1996) Changes in expression of transforming growth factor beta mRNA isoforms in patients undergoing tamoxifen therapy. *Br J Cancer* **74**: 474–478

Perry RR, Kang Y and Greaves BR (1995) Relationship between tamoxifen-induced transforming growth factor β1 expression, cytostasis and apoptosis in human breast cancer cells. *Br J Cancer* **72**: 1441–1446